# Mapping brain endophenotypes associated with idiopathic pulmonary fibrosis genetic risk

**DOI:** 10.1016/j.ebiom.2022.104356

**Published:** 2022-11-19

**Authors:** Ali-Reza Mohammadi-Nejad, Richard J. Allen, Luke M. Kraven, Olivia C. Leavy, R. Gisli Jenkins, Louise V. Wain, Dorothee P. Auer, Stamatios N. Sotiropoulos

**Affiliations:** a National Institute for Health Research (NIHR) Nottingham Biomedical Research Centre, Queens Medical Centre, Nottingham, United Kingdom; b Sir Peter Mansfield Imaging Centre & Mental Health and Clinical Neurosciences, School of Medicine, University of Nottingham, Nottingham, United Kingdom; c Department of Health Sciences, University of Leicester, Leicester, United Kingdom; d National Heart and Lung Institute, Imperial College London, London, United Kingdom; e Department of Interstitial Lung Disease, Royal Brompton and Harefield Hospital, Guys and St Thomas' NHS Foundation Trust, London, United Kingdom; f National Institute for Health Research (NIHR) Leicester Respiratory Biomedical Research Centre, Glenfield Hospital, Leicester, United Kingdom

**Keywords:** Idiopathic pulmonary fibrosis (IPF), Magnetic resonance imaging (MRI), Neuroimaging, Genetics, Lungs

## Abstract

**Background:**

Idiopathic pulmonary fibrosis (IPF) is a serious disease of the lung parenchyma. It has a known polygenetic risk, with at least seventeen regions of the genome implicated to date. Growing evidence suggests linked multimorbidity of IPF with neurodegenerative or affective disorders. However, no study so far has explicitly explored links between IPF, associated genetic risk profiles, and specific brain features.

**Methods:**

We exploited imaging and genetic data from more than 32,000 participants available through the UK Biobank population-level resource to explore links between IPF genetic risk and imaging-derived brain endophenotypes. We performed a brain-wide imaging-genetics association study between the presence of 17 known IPF risk variants and 1248 multi-modal imaging-derived features, which characterise brain structure and function.

**Findings:**

We identified strong associations between cortical morphological features, white matter microstructure and IPF risk loci in chromosomes 17 (*17q21.31*) and 8 (*DEPTOR*). Through co-localisation analysis, we confirmed that cortical thickness in the anterior cingulate and more widespread white matter microstructure changes share a single causal variant with IPF at the chromosome 8 locus. Post-hoc preliminary analysis suggested that forced vital capacity may partially mediate the association between the *DEPTOR* variant and white matter microstructure, but not between the *DEPTOR* risk variant and cortical thickness.

**Interpretation:**

Our results reveal the associations between IPF genetic risk and differences in brain structure, for both cortex and white matter. Differences in tissue-specific imaging signatures suggest distinct underlying mechanisms with focal cortical thinning in regions with known high *DEPTOR* expression, unrelated to lung function, and more widespread microstructural white matter changes consistent with hypoxia or neuroinflammation with potential mediation by lung function.

**Funding:**

This study was supported by the NIHR Nottingham Biomedical Research Centre and the UK Medical Research Council.

**Research in context**

**Evidence before this study**

Idiopathic pulmonary fibrosis (IPF) is a condition in which the lungs become scarred (fibrosed). Although IPF primarily affects the lungs, co-occurrence with impaired brain function, such as cognitive decline, increased risk of neurodegenerative disorders, cerebrovascular accidents, depression, and anxiety have been reported. The nature of this association is unclear and no post-mortem or in-vivo investigations have been performed to explore this directly. At the genetic level, 17 regions of the genome have been found to drive the genetic risk for IPF.

**Added value of this study**

Using previously identified IPF genetic variants and neuroimaging-derived features from 32,431 participants available through the UK Biobank, we performed a brain-wide association study for IPF risk variants. We identified brain endophenotypic associations with two IPF risk associated genetic variants, one in chromosome 17 and one in chromosome 8. In particular, the presence of the IPF risk variant at the *DEPTOR* gene locus on chromosome 8, was associated with focal cingulate cortical thinning and more widespread changes in white matter microstructure. The cortical association signature was observed in regions with known *DEPTOR* expression. The white matter findings may be mediated by forced vital capacity (measure of impaired lung function), hinting to distinct direct (cingulate) and indirect (subcortical) brain effects of *DEPTOR* risk alleles.

**Implication of all the available evidence**

The reported brain-wide IPF risk association patterns reveal two potential mechanisms that may explain the known association of IPF and brain disorders: (i) direct and focal, thought to be related to paralimbic mTOR dysregulation and (ii) indirect and widespread, in keeping with secondary effects from impaired lung function, such as hypoxia. Taken together, these data support the hypothesis that genetic risk profiles may explain some of the observed comorbidity of IPF and brain disorders.

## Introduction

Idiopathic pulmonary fibrosis (IPF) is a serious chronic lung disease of unknown aetiology affecting around five million people worldwide.^1^ It is characterised by shortness of breath and causes the lung tissue to become stiff and scar over time. As a result, the lung parenchyma is replaced with a dense extracellular matrix.^2^ IPF is typically diagnosed in middle-age, progression and survival is quite variable between affected people,^3^ but progressive fibrosis leads ultimately to death with a median survival of 3–5 years after diagnosis.^4^

Although the pathogenesis of IPF is incompletely understood, there is a growing body of evidence that multiple risk factors including ageing, genetic alterations, and environmental factors, such as cigarette smoke exposure, contribute to disease risk. Genome-wide association studies (GWAS) have identified a number of loci associated with increased genetic risk in IPF.^5^, ^6^, ^7^, ^8^ Although IPF is considered a disease limited to the lung, the mechanisms responsible for the development of fibrosis in the lung are often shared with fibrosis in other organs^9^ which may explain associated comorbidities.^10^^,^^11^ Whilst there are recognised associations between organs, such as lung and liver fibrosis,^12^ others are less well-known and understood. For instance, co-occurrence of IPF with impaired brain function has been reported,^13^^,^^14^ however, no study so far has explicitly explored links between IPF and specific brain features.

There has been scattered evidence in the literature for brain-lung relationships, across the spectrum of respiratory disorders.^15^, ^16^, ^17^, ^18^, ^19^, ^20^ Pulmonary fibrosis in particular has been clinically associated with cognitive decline,^13^^,^^21^ increased risk of neurodegenerative disorders,^14^^,^^21^ cerebrovascular accidents,^22^ and a high prevalence of anxiety (30–50%) and depression (about 20–30%).^23^ However, the mechanisms linking IPF to brain changes and dysfunction are poorly understood and likely multifactorial; including either direct shared pathomechanisms that result in fibrosis and scar development in the lung and to altered brain development and accelerated neurodegeneration or indirect sequelae of brain injury resulting from fibrosis-associated hypoxic tissue damage. To the best of our knowledge, no post-mortem or in-vivo investigations have been performed to explore these directly.

Neuroimaging can enable unique insight into the nature of the association between IPF and brain dysfunction due to its remarkable sensitivity to subtle structural and functional brain changes. It can reveal topographical and tissue-specific brain signatures linked to genetic risks of primary brain disorders or comorbid brain health in systemic disease. Brain imaging has been successfully used to provide intermediate phenotypes (sometimes referred to as endophenotypes) to assist GWAS studies in brain disorders^24^ or to explore the link between risk and clinical phenotype.^25^ Recent advances with population-level data and tools for systematic extraction of quantitative brain imaging-derived phenotypes (IDPs)^26^ permit the investigation of associations between IDPs and genetic risk profiles to discover novel intermediate brain phenotypes; these may not be directly linked to expressed behaviours but reflect altered cellular and molecular functions.^24^^,^^27^ This approach is particularly powerful to understand brain comorbidities without robust prior mechanistic or regional knowledge and with low or non-specific clinical manifestations.

In this study we used a brain-wide exploratory approach, capitalising on population-level datasets available through the UK Biobank.^26^^,^^28^ Similar to phenome-wide association studies, which examine the associations between specific genetic variants and a wide array of phenotypes across the genome, we performed a brain-wide association study (BWAS) to examine the association of genetic variants linked with the risk of IPF (Supplementary Table S1) across a large array of 1248 multimodal imaging-derived phenotypes (IDPs) (Supplementary Table S2), which capture morphological, (micro)structural, and functional brain features. Using data from 32,000 participants, we identified associations between IPF risk variants in chromosome 8 and 17 with focal cortical morphological changes and more widespread white matter microstructure alterations in the brain. We further explored these findings in a co-localisation analysis with IPF susceptibility and in mediation analysis, using a measure of pulmonary function as a potential mediator in the IPF variants-IDPs associations. Taken together, these analyses allowed us to reveal and characterise links between brain cortical and subcortical features and the presence of lung fibrosis risk genetic variants.

## Methods

### Study cohort selection from the UK Biobank

**Fig. 1:**
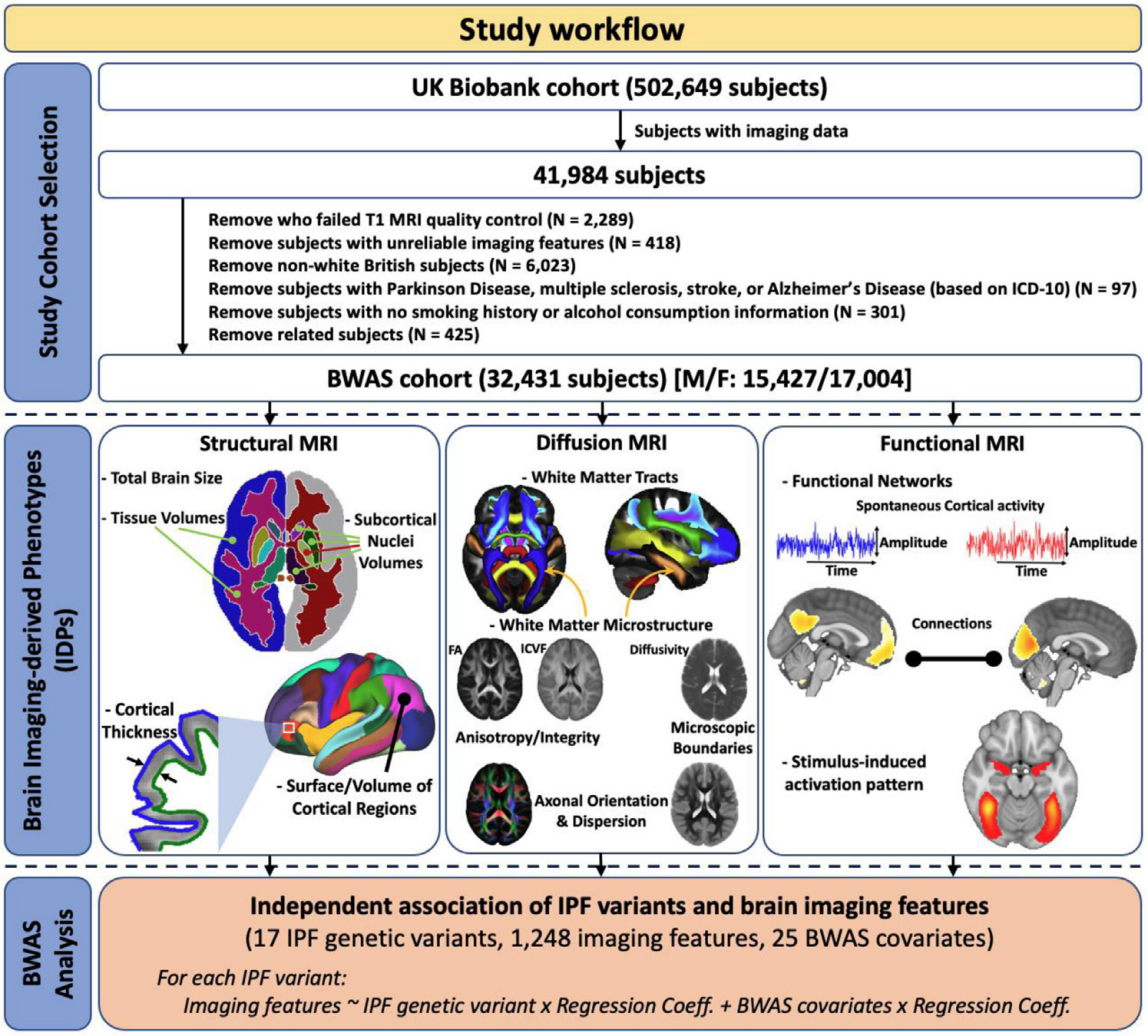
**The workflow schematic for data selection, neuroimaging-derived feature extraction, and subsequent BWAS analysis.** MRI, magnetic resonance imaging; BWAS, brain-wide association study; IPF, idiopathic pulmonary fibrosis; FA, fractional anisotropy; ICVF, intra-cellular volume fraction; MS, multiple sclerosis. The list of BWAS covariates is age, sex, age^2^, age × sex, age^2^ × sex, standing height, weight, diastolic and systolic blood pressures, the top 10 principal components of the genetic matrix, smoking status, and alcohol consumption.

Neuroimaging and genetic datasets from the population-level UK Biobank resource were used.^26^^,^^28^ From the entire UK Biobank cohort (∼500,000 participants), we derived our study cohort of 32,431 participants (Fig. 1), based on *a priori* selection criteria. We considered participants that had: i) Neuroimaging (magnetic resonance imaging—MRI) and genetic data; Based on the UK Biobank data released in April 2020, only 41,984 subjects had brain imaging data. ii) Good-quality anatomical MRI (T_1_-weighted) data; We excluded 2289 subjects because of poor structural imaging data (for example, due to having incomplete brain coverage, or having very severe MRI artefacts).^29^ iii) Reliable neuroimage processing and features extracted; IDP values that were greater/less than five times the standard deviation from the cohort-mean IDPs were considered technical outliers and thus removed. We removed 418 subjects due to unreliable imaging features in more than 10 IDPs. iv) White European ethnic background, to keep ethnicity consistent; Based on the self-reported ethnicity or the similarity of their genetic ancestry, 6023 participants were non-white European and were excluded (Data-Field 22006). v) No major neurological conditions reported; To reduce bias from major IDP variation of no interest, we excluded neurological disorders (Multiple Sclerosis, Stroke, Parkinson's Disease, or Alzheimer's Disease) with known large effects on imaging features, through the underlying disease or its medication. Based on the main and secondary ICD-10 (Data-Field 41202 and 41204), 97 individuals were excluded from the analysis due to been diagnosed with either Parkinson's Disease (code G20, G21, G22, G210–G214, G218, G219), Multiple Sclerosis (code G35), Alzheimer's Disease (code G30, G300, G301, G308, G309), or had a Stroke (code G46, G460–G468, I60–I64, I600–I616, I618–I621, I629–I636, I638–I639). vi) Available information on smoking status and alcohol drinking; we excluded 301 individuals that did not have smoking status (Data-Field 20116), alcohol drinker status (Data-Field 20117), or alcohol intake frequency (Data-Field 1558) information available. vii) No relationship to other study participants; we removed 425 subjects that were family-related to other individuals in the cohort with a KING kinship coefficient ≥ 0.0884^30^ (from each group of related individuals we chose only one representative, forming a maximally unrelated subset—relatedness file downloaded from UK Biobank). viii) Consistent genotype and self-reported sex (Data-Field 31 and 22001). Our selected study cohort included individuals aged between 45 and 82 years (mean = 64.2, std = 7.5 years) at the time of imaging.

### Imaging data and imaging-derived phenotypes

A large set of neuroimaging features (also called imaging-derived phenotypes—IDPs) extracted from all available imaging modalities using a standardised processing pipeline^26^^,^^29^ was provided and obtained by the UK Biobank. Modalities included: i) Structural MRI (T_1_-weighted, T_2_-weighted, and susceptibility-weighted imaging (SWI)) that provides anatomical and morphological information; ii) Diffusion MRI (dMRI) that reflects white matter microstructural complexity and integrity derived from two models, the diffusion tensor imaging (DTI) and neurite orientation dispersion and density imaging (NODDI); and iii) Functional MRI (fMRI), which indirectly reflects neural activity and cortical connectivity during task-free (resting-state) and task-based conditions. To minimise redundancy in the original set of IDPs, we considered a reduced set of N = 1248 IDPs for each participant, representing all the above modalities and features, but minimising overlap (for instance we kept only one set of cortical volumes, functional connectivity, and white matter microstructure rather than all flavours of the same features provided) (full list in Supplementary Material/Table S2). Following,^26^^,^^31^ and to reduce the effect of potential outliers and improve the accuracy of associations, we applied rank-based inverse Gaussian transformation (quantile normalization) on all IDPs.

### Imaging de-confounding

As imaging-derived features can be affected by non-biological parameters, we de-confounded them following the recommendations in.^31^^,^^32^ In total, 45 features were used as the main imaging confounds set for this study. The imaging confound variables included: a) Volume-to-volume head motion, the micro-movements of the head between two consecutive volumes (time-points) in dynamic imaging (resting-state fMRI and task fMRI), averaged across all volumes. b) Head size scaling, defined as the volumetric scaling from the T1 head image to a template MNI152 standard space image. c) Head position within the scanner, described by the scanner lateral (X), transverse (Y), and longitudinal (Z) brain position, and the scanner table position, as data quality is highly dependent on the exact location of the head and the radiofrequency receive coil in the scanner. d) The MRI scanning site, as the data have been collected from three UK Biobank imaging sites. Even if UK Biobank uses identical scanner hardware and software for all three sites, there are still subtle differences between them. e) Date-related drift. As suggested by,^31^^,^^32^ there are slowly changing drifts in the data. To capture those, we first generated a matrix of Subjects × IDPs. To overcome any missing data, this matrix was imputed using a low-rank matrix imputation algorithm.^33^ To remove outliers, we applied a median-based outlier removal (discarding values greater than five times the median absolute deviation from the overall median). The matrix was then temporally regularized (with respect to the scan date—Data-Field 53) with spline-based smoothing. Then, after a PCA, the top 10 components were kept to reflect the primary modes of slowly changing drifts in the data and be used as imaging confounds.

As suggested in,^26^^,^^31^^,^^34^ for these imaging confounds variables we generated augmented versions as well (for example quantile normalisation, outlier removal, and centred squared versions of these imaging confounds). Supplementary Table S3 provides a list of all the imaging confounding variables used. A general linear model of the original IDPs against these imaging confounding regressors allowed us to regress out their effects on the IDPs and obtain de-confounded IDPs.

### Genetic data pre-processing

For our study cohort, we used the previously described imputed UK Biobank genotype data released in November 2020 (Data-Field 22828, ver.3 of imputed data).^28^ In our previous work^5^^,^^35^ seventeen loci have been associated (P-value < 5 × 10^−8^) with increased genetic risk in IPF (Supplementary Table S1), from a large meta-GWAS study, using three independent IPF case–control collections (6, 7, and 8—2668 IPF cases and 8951 controls) and a replication analysis performed in two independent studies (5 and 36—1456 IPF cases and 11,874 controls). All 17 IPF variants (single-nucleotide polymorphisms—SNPs) were present in the UK Biobank imputed data and were used for subsequent analyses.

### Brain-wide association study (BWAS) against IPF variants

We tested the association between each of the 17 IPF variants and each of the 1248 IDPs across 32,431 individuals. For each IPF variant, separate analyses were performed using a general linear model (PLINK v2.0 software^37^). An additive genetic effect was assumed (Supplementary Figure S1) and we adjusted for certain BWAS covariates. The allele associated with increased risk of IPF was chosen as the coded allele. We tested for statistical significance of the observed genetic association after applying Bonferroni correction for multiple comparisons (P-value < 0.05/(17 × 1248)). The number of subjects with available data for each IDP varied from a minimum of 22,892 to a maximum of 32,431.

### Brain-wide association study (BWAS) covariates

The de-confounded IDPs were used along with single-nucleotide polymorphism (SNP) data in the BWAS model to identify brain-wide Imaging-Genetics associations. As suggested in,^32^ we used the following variables as covariates in the BWAS model (see also Supplementary Table S3): i) Demographic measures. We used age (the difference between the date of birth and scanned date, Data-Fields 34, 52, and 53), sex (Data-Field 31), age^2^, age × sex, age^2^ × sex. ii) Body measurements. We used standing height (Data-Field 50), and weight (Data-Field 21002) as covariates. Any missing value in height and weight data was substituted by the imputation (using the R package “mice”^38^) of height and weight values prior to imaging stages and the second imaging session (if available). Diastolic and systolic blood pressures were measured several times during the imaging visit using automated and manual blood pressure reading devices (Data-Fields 4079, 4080, 79, 80). We averaged the automated and manual blood pressure readings. The missing values were replaced with the imputed version of these readings from other sessions. iii) Genetic information. We used genetic matrix principal components (the top 10 principal components, Data-Field 22009), as covariates to adjust for population stratification. iv) Lifestyle measures. Measures related to smoking and alcohol consumption were used as covariates in the model. For smoking, we generated an ‘ever smoker’ covariate. To derive this covariate, we used ‘smoking status’ (Data-Field 20116). This field summarises the current/past smoking status of the participants. For the participants who have clearly answered this question at the imaging visit, that answer is used for their status. Otherwise, we used their answer for their first repeat assessment or the initial assessment visit. Then, previous and current smokers were grouped together to make an ‘ever smoker’ covariate (1: current/previous, 0: never). For alcohol consumption, we derived a variable that reflects the number of alcohol units per week for each participant. To do that, for all participants who answered they consumed alcohol (Data-Field 20117), we used their alcohol intake frequency (Data-Field 1558) and their intake for different types of drink per week or per month using standard drink sizes (Data-Fields 1568, 1578, 1588, 1598, 1608, 5364, 4407, 4418, 4429, 4440, 4451, 4462). Then, for each type of drink, we applied a standardized number of UK alcohol units to be able to estimate the number of units of alcohol consumption per week for each participant.^39^

The BWAS covariates were de-meaned and unit-variance normalised. In total, 25 variables were used as covariates in the BWAS models (Supplementary Table S3). When multiple instances of a covariate were available, the values recorded at the imaging visit were used.

### Co-localisation analysis: identification of shared causal variants

For the genetic variants that were found to be associated with brain IDPs, we performed co-localisation analysis using the COLOC method^40^ (details in Supplementary Material). This allowed us to explore whether the associations between IPF susceptibility (trait 1) with the brain phenotype/IDPs (trait 2) were likely due to the same causal variant (assuming it has been tested and there is only one causal variant). The IPF susceptibility GWAS data were obtained from a large meta-GWAS study of IPF.^5^ For the BWAS data, we selected the IDPs that corresponded to the most significant associations (that survived Bonferroni) in BWAS. SNPs within 1 Megabase (Mb) window from each side of the lead SNP were included. For trait 2, we selected the same SNPs (as used for trait 1) within ± 1 Mb window from the lead SNP.

### Mediation analysis

To explore whether associations between neuroimaging features (IDPs) and presence of IPF risk genetic variants, as derived from the BWAS regression model, were indirect effects from subclinical IPF risk manifestation, we undertook mediation analysis focusing on basic lung function that could plausibly affect brain health. We used forced vital capacity (FVC–best measure, Data-Field 20151), a cumulative measure of lung function (subclinical phenotype),^41^ as an exploratory mediator (Supplementary Material/Figure S2). We constructed a mediation pathway between a single independent variable (IPF genetic risk *X*), a single mediator (FVC *M*), and a single dependent variable (brain IDPs *Y*). This aimed to quantify the direct and indirect relationships between IPF risks, IDPs, and FVC. The direct and indirect standardized effects were calculated using multivariate-adjusted linear regression analysis and a Sobel test^42^ was used to determine whether the indirect effect of the SNP on IDPs through FVC was significant. Only IDPs that were found to associate with potentially causative genetic risk in the previous analyses were used in the mediation models.

### Statistics

The statistical software, threshold, sample size, inclusion/exclusion criteria, and methods of each analysis are described in the sections above. All the analysis was conducted using PLINK v2.0,^37^ MATLAB 2019b, and R version 3.5.1.

### Ethics

No new data were collected from this study. We used publicly-available data from the UK Biobank (under Project 43822). The UK Biobank has approval from the North West Multi-centre Research Ethics Committee (MREC) to obtain and disseminate data and samples from the participants (http://www.ukbiobank.ac.uk/ethics/), and these ethical regulations cover the work in this study. Written informed consent was obtained from all participants.

### Role of the funding source

Funding sources played no role in study design; collection, analysis, or interpretation of the data; writing of the report, or in submission of this paper for publication.

## Results

### Brain imaging-derived phenotypes associate with IPF genetic variants

**Fig. 2:**
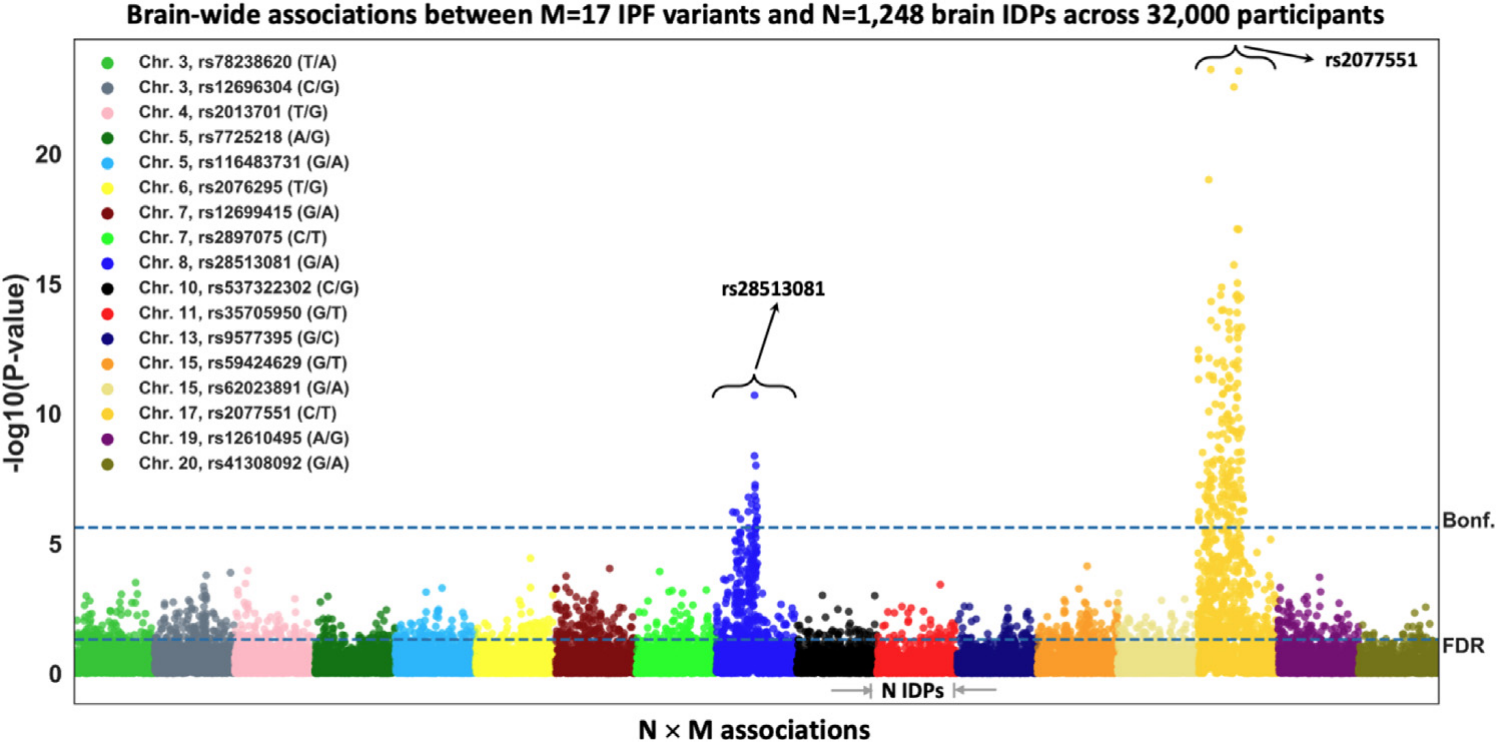
**Manhattan plot of the BWAS outcomes of 17 known IPF variants using UK Biobank imaging cohort (S = 32,431).** Each of the IPF variants is coded by a different colour and within each of these colour groups, associations with the 1248 brain IDPs, derived from neuroimaging, are depicted. For each association between a SNP with an IDP, its −log10(P-value) is plotted (i.e., M × N = 17 × 1248 associations are shown). The dashed horizontal line indicates the brain-wide level of significance, i.e., FDR (false discovery rate—bottom line, corresponding to FDR-corrected P-value = 0.05) and Bonferroni correction across M × N = 21,216 tests (top line, P-value < 2.36 × 10^−6^).

We explored brain-wide associations of the 17 IPF genetic variants against 1248 brain IDPs across 32,431 unrelated white British participants in the UK Biobank study. Fig. 2 shows Manhattan plots summarising the results for all 17 × 1248 associations. Two of the 17 IPF variants (rs28513081 (G/A)—*DEPTOR* gene in chromosome 8—and rs2077551 (C/T)—*17q21.31* in chromosome 17) were found to be significantly associated with brain IDPs. The number of associations reaching the Bonferroni-corrected threshold for statistical significance (P-value < 2.36 × 10^−6^) were 19 and 174 for the chromosome 8 and chromosome 17 IPF variants, respectively (giving in total 184 brain IDPs—full list in Supplementary Table S4).

### Presence of IPF risk genetic variant associates with cortical morphology and white matter microstructure

**Fig. 3:**
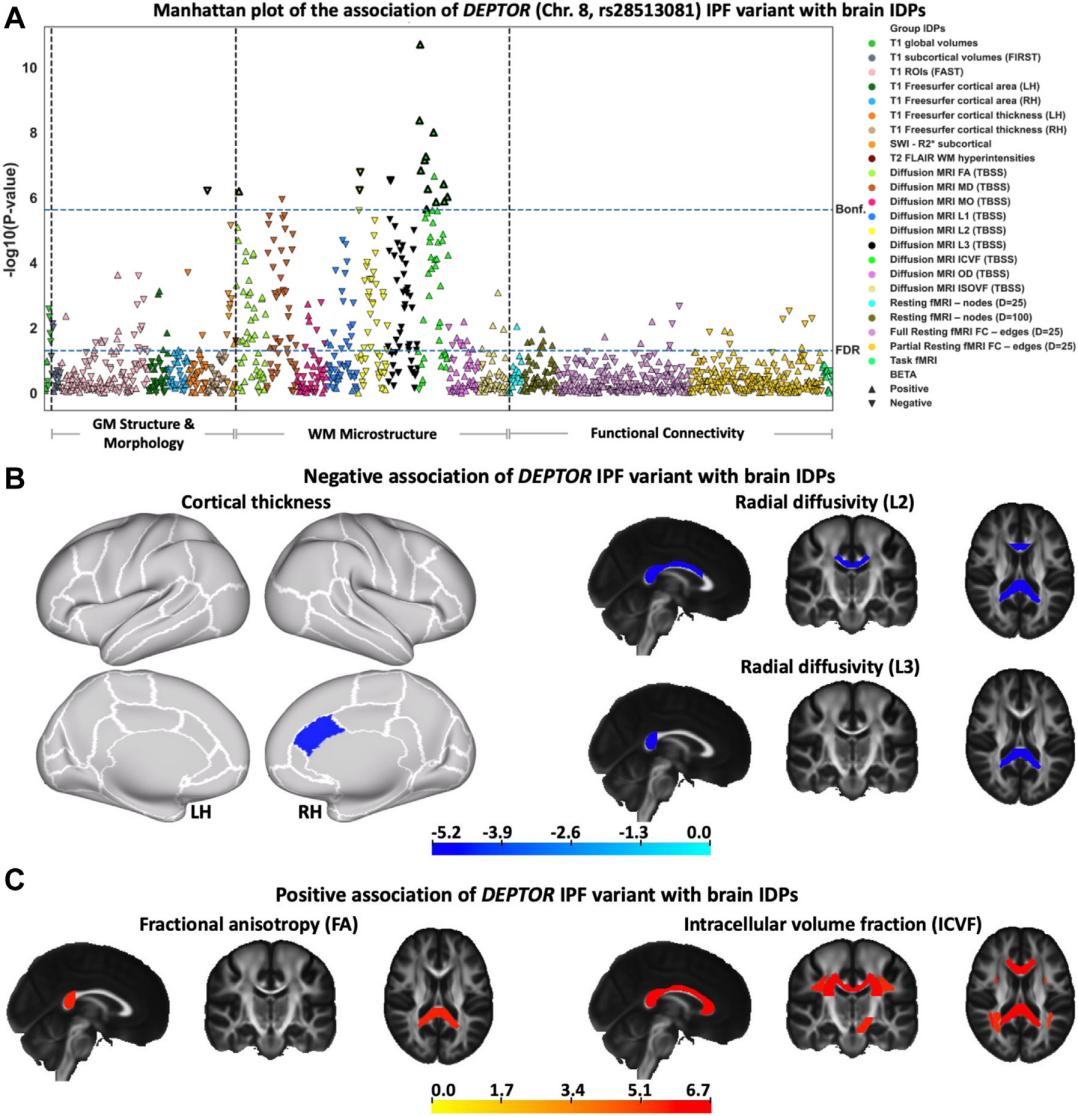
**The association of the *DEPTOR* IPF variant with brain IDPs.(a)** BWAS analysis of rs28513081 (*DEPTOR* gene in chromosome 8). Points are coloured based on the group IDPs. The IDPs included in each group of features are available in Supplementary Table S2. Triangles and inverted triangles are used to show the sign of the BETA in the BWAS. The dashed lines indicate the level of significance, i.e., FDR and Bonferroni thresholds (P-value < 2.36 × 10^−6^). The vertical dashed lines separate the IDPs belongs to the grey matter (GM) structure and morphology, WM microstructure, and functional connectivity. A positive (negative) BETA means that a trait positively (negatively) associated with the presence of the *DEPTOR* IPF risk variant. The triangles with bold outlines in **(a)** demonstrate IDPs that co-localise with IPF susceptibility as found by co-localisation analysis presented in Fig. 4. **(b)** Negative and **(c)** positive associations of neuroimaging features (IDPs) with IPF variant in rs28513081 (*DEPTOR* gene in chromosome 8). T-stat maps of the associations are shown for the most significant IDPs (that survived Bonferroni correction) associated with the fibrosis variant. Positive t-stats correspond to positive association with the presence of IPF risk variant in chromosome 8. T1 Freesurfer-extracted cortical thickness, diffusion MRI L2, and diffusion MRI L3 negatively associated with the *DEPTOR* IPF variant. Diffusion MRI FA and diffusion MRI ICVF positively associated with the *DEPTOR* IPF variant. In the cortical thickness map, the white contours show the outline of the regions in Desikan-Killiany–Tourville (DKT) atlas and top and bottom panels show the lateral and medial view of the brain, respectively. In other spatial maps, the standard HCP1065 FA image is used as background (left is right). LH, Left hemisphere; RH, Right hemisphere.

Within the brain IDPs, we explored which categories of features demonstrated associations with the genetic data. For both chromosome 8 and chromosome 17 IPF variants, we found that the associations were driven by brain morphological and microstructural features, in cortex and white matter, and interestingly no associations were found with functional features extracted from functional MRI (e.g., functional networks and connectivity) (Fig. 3a and Supplementary Figure S3a).

Fig. 3b–c shows spatial maps of the 19 IDPs associated with the *DEPTOR* IPF variant that survived Bonferroni correction, along with the sign and magnitude of the association (t-stat). Cortical thickness in the caudal anterior cingulate was negatively associated with IPF risk (lower thickness ↔ presence of IPF risk variant in chromosome 8). A number of white matter (WM) IDPs (mostly reflecting anisotropy, such as the fractional anisotropy (FA) and the NODDI intracellular volume fraction (ICVF), and radial diffusivity indices) of major fibre bundles (corpus callosum, superior longitudinal fasciculus, and corona radiata) were also associated with the *DEPTOR* IPF variant. These IDPs represent WM integrity and microstructure within these bundles. ICVF and FA correlated positively with the presence of IPF risk variant (Fig. 3c), whilst radial diffusivity values correlated negatively (Fig. 3b) (i.e. higher ICVF, FA, and lower diffusivity ↔ presence of IPF variant in chromosome 8).

We also observed strong associations of brain IDPs with IPF genetic risk for the rs2077551 (chromosome 17) variant, where 174 associations with brain IDPs were found (Supplementary Figure S3). The modalities of associated IDPs and direction of changes were similar to those reported for the *DEPTOR* IPF variant. Significant associations with the rs2077551 (chromosome 17) variant included indices of white matter integrity and microstructure (such as the fractional anisotropy (FA), the NODDI ICVF, and mean diffusivity (MD)), and cortical morphological measures (such as cortical thickness and cortical area, SWI, and T1 global/subcortical/regions of interest (ROIs) volumes), with the most significant association corresponding to “cortical surface area of fusiform—right hemisphere (RH)”. No functional measures associated with this IPF variant either. Presence of IPF risk variant in chromosome 17 was associated with increased cortical thinning in certain areas (e.g., inferior and middle temporal regions), with lower diffusivities in white matter (Supplementary Figure S3b), and higher intra-cellular volume fraction/anisotropy in white matter (in bundles including corpus callosum, corona radiata, and association tracts). Conversely to the pattern of associations identified with the *DEPTOR* variant, positive associations of cortical surface area with the chromosome 17 IPF variant were also found (e.g., fusiform and lingual areas) (Supplementary Figure S3c).

**Fig. 4:**
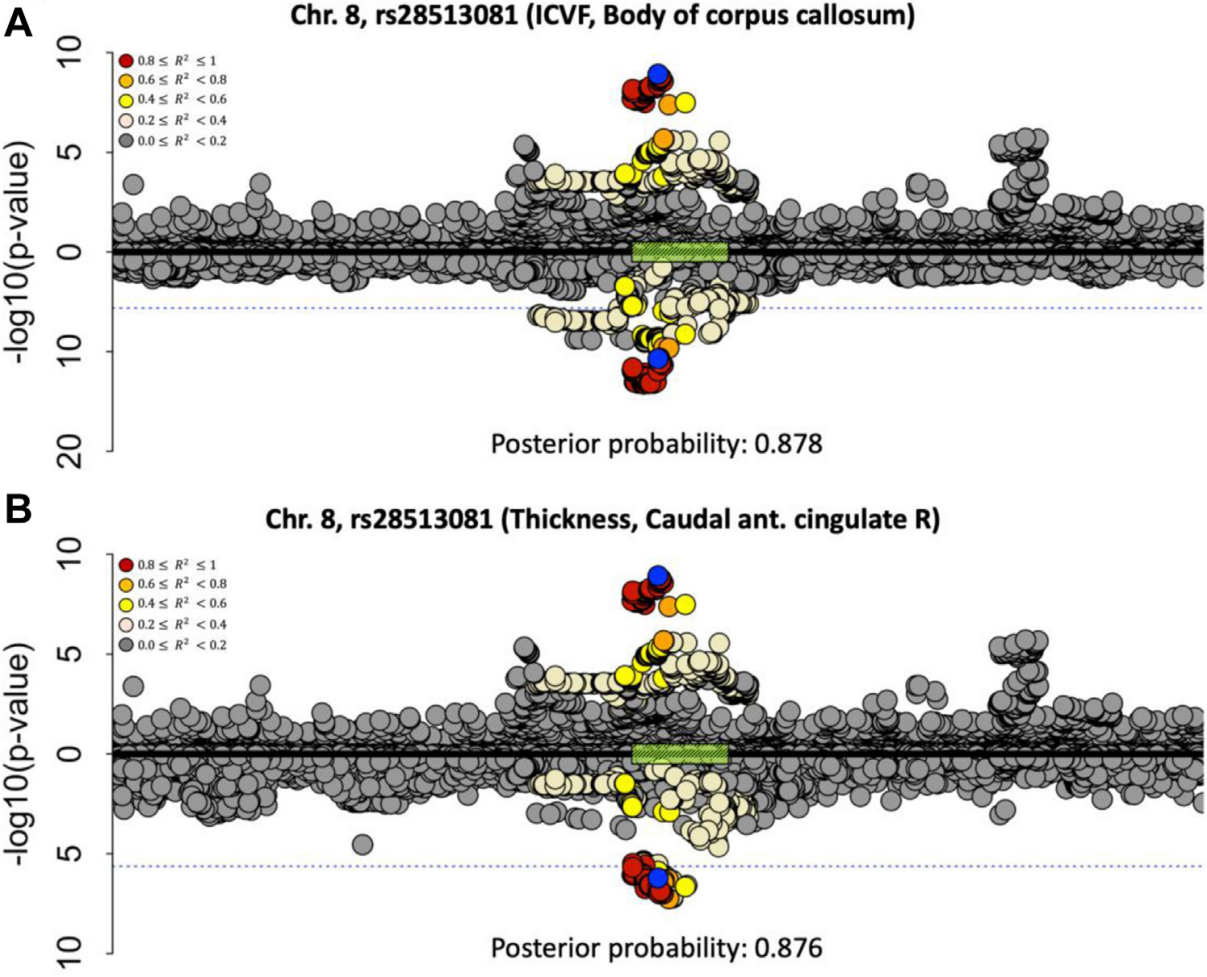
**Co-localisation of the two most significantly associated IDPs with chromosome 8 (region around the *DEPTOR* gene), against IPF risk.(a)** Against a white matter microstructure feature (ICVF of the body of corpus callosum), **(b)** Against a cortical feature (cortical thickness of the caudal anterior cingulate RH). Chromosome 8: 119,934,133–121,934,069 build x-axis for both plots. Each point represents a genetic variant with chromosomal position on the x-axis and −log(P-value) on the y-axis. The GWAS data for IPF risk is presented above the x-axis, and the GWAS data for the IDP is shown below the x-axis. The sentinel variant from the IPF GWAS is shown in blue and other variants are coloured by their linkage disequilibrium with the IPF GWAS sentinel. The dashed blue lines indicate the Bonferroni threshold (2.36 × 10^−6^). The green box on the x-axis demonstrates the position of the *DEPTOR* gene. To show the GWAS data for IPF risk, we used the variant summary data of the discovery stage (including three independent IPF case–control collections (named the UK, Chicago, and Colorado studies comprised up to 2668 IPF cases and 8951 controls) of the study by Allen et al.^5^ The R^2^ is calculated from the actual cohort.

To explore whether the association of IPF genetic risk with the brain IDPs was likely due to the same causal variant, we performed co-localisation analysis for the IDPs that were found to be significantly associated with IPF variants. Seventeen of the nineteen significant IDP associations for the chromosome 8 IPF locus showed statistical evidence through co-localisation of a likely shared causal variant with the IPF association (posterior ≥ 0.8^43^). Two representative examples, one for a white matter IDP and one for a cortical IDP are shown in Fig. 4. The co-localisation plots for the remaining IDPs are shown in Supplementary Figures S4 and Supplementary Table S5. For the chromosome 17 locus, co-localisation analysis was inconclusive due to the complex structural variation in the region that results in a large number of highly correlated SNPs across 1.5 Mb.^44^

Taken together, the above results provide strong evidence—through complementary analyses—of a potentially causal association between the *DEPTOR* (chromosome 8) IPF variant and 17 brain IDPs, representing cortical morphology and white matter microstructure. We kept these 17 IDPs for subsequent analysis, indicated by the triangles with bold outlines in Fig. 3a.

### IDP associations with the *DEPTOR* variant may be partially mediated by lung function

To explore potential mechanisms underlying the identified associations, we conducted a mediation analysis. Given that IPF is a restrictive lung disease characterised by impaired lung capacity, we used forced vital capacity (FVC), which is used for IPF diagnosis and characterisation,^45^ to examine whether the associations between genetics and neuroimaging features are mediated through variation in lung function irrespective of underlying pathology. We hypothesised that the effect of IPF genetic risk on brain IDPs is indirect via its influence on FVC (Supplementary Figure S2).

**Fig. 5:**
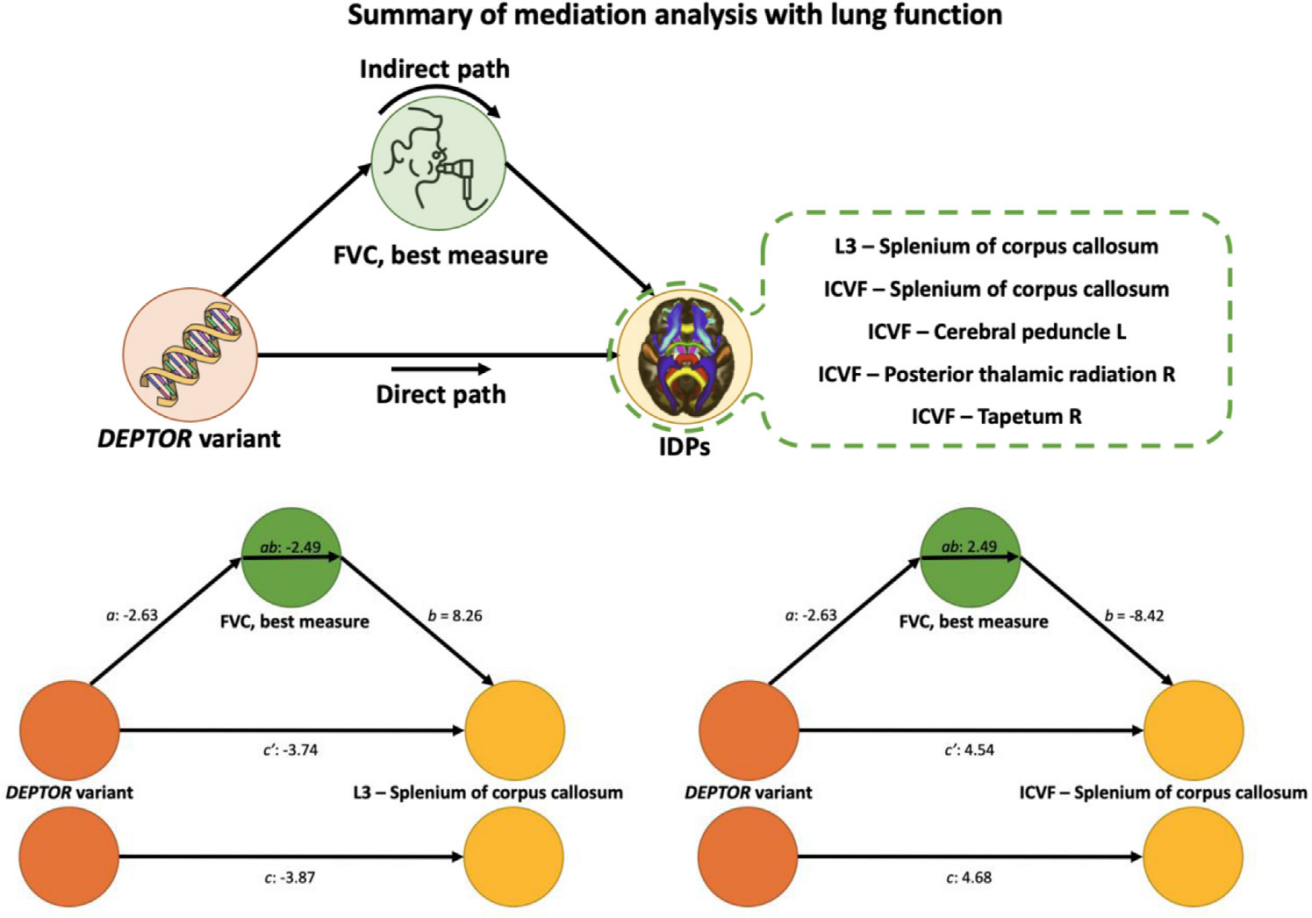
**Secondary analysis. List of the brain IDPs that associate with the *DEPTOR* gene variant via FVC as a mediator and two examples of the mediation analyses results.** The plots demonstrate the direct, indirect (mediated by FVC—best measure), and total effect paths from the *DEPTOR* variant to brain IDPs. The bottom left path diagram shows the mediation through FVC for *DEPTOR* which is negatively associated with the diffusion MRI L3 (splenium of corpus callosum). The bottom right path diagram depicts the mediation through FVC for *DEPTOR* which is positively associated with the diffusion MRI ICVF (splenium of corpus callosum). The Z-scores of the regression coefficient are shown for paths *a*, *b*, *c*, *ab*, and *c’*.

Mediation analysis showed that the association between the *DEPTOR* IPF variant and the anterior cingulate cortical thickness was not mediated by lung function, as captured by FVC (Fig. 5). On the other hand, some of the white matter microstructure measures exhibited a different trend. For five of them, representing microstructure in the corpus callosum, cerebral peduncles, and thalamic radiations, the association with the IPF risk variant was partially mediated by FVC (Fig. 5, full results in Supplementary Figure S5/Table S6), albeit results did not survive multiple comparison correction using a Bonferroni threshold (0.05/17) (uncorrected P-values ranging from 0.0128 to 0.0425). Even if only reaching nominal significance and therefore only providing preliminary evidence, the direct path for these associations was attenuated in line with potential partial mediation by FVC. As an example, the details of direct and indirect paths of a positively and negatively associated IDP are shown in Fig. 5. Interestingly only subcortical (white matter) brain IDPs showed potential (but weak) FVC mediation effects of their associations with IPF risk variants.

**Fig. 6:**
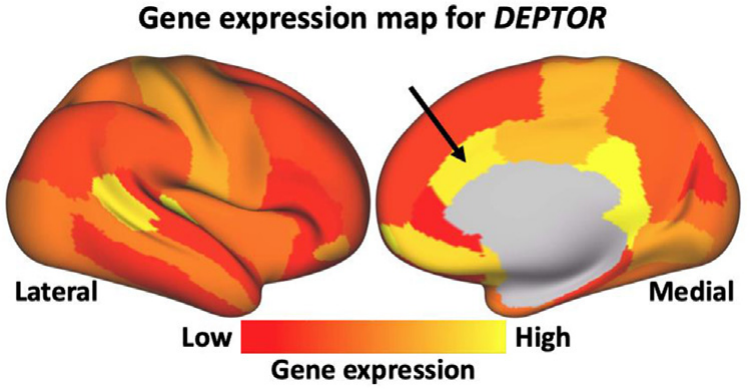
**The group-level gene expression patterns for chromosome 8 (*DEPTOR* gene) projected on an inflated cortical surface.** The colour bar shows the gene-expression level. Red regions show areas where the gene of interest is highly expressed, whereas yellow regions indicate low expression values. The black arrow denotes the caudal anterior cingulate, where reduced cortical thickness has been found to associate with the IPF *DEPTOR* variant.

The lack of any mediation through FVC in the case of anterior caudate cortical thickness can hint to a likely direct gene effect that is limited to the anterior caudal cingulate cortex. To corroborate the likely direct nature of this effect, we computed a bulk gene expression map of the *DEPTOR* gene, using the Allen Human Brain Atlas (AHBA) database^46^ and the same cortical surface parcellation as the one used in our IDP extraction pipeline (details in Supplementary Material). As shown in Fig. 6, the caudal anterior cingulate region (denoted by the arrow), i.e., the area where reduced cortical thickness was found to associate with the presence of the *DEPTOR* IPF variant, has a relatively high expression level for the *DEPTOR* gene. That provides evidence of biological plausibility that the observed association between IPF risk gene variant and IDP changes was detected in a cortical region where the risk gene is indeed strongly expressed.

## Discussion

We undertook a large brain-wide multi-modal imaging and genetic association study in 32,000 participants of the UK Biobank to establish brain endophenotypes of IPF genetic susceptibility. Strong associations with a likely shared causal variant were found for several of the 1248 studied brain imaging-derived phenotypes (IDPs) for two of the 17 IPF risk associated variants on chromosomes 8 and 17. Co-localisation provided additional evidence that the associated neuroimaging features and IPF share a single causal variant at the chromosome 8 locus. The association pattern consists of focal cingulate cortical thinning and more widespread microstructural alterations in major white matter tracts, such as the corpus callosum and corona radiata. Furthermore, we suggest potential partial mediation of the subcortical IDP–*DEPTOR* variant associations by lung function in keeping with an indirect effect such as systemic hypoxia or inflammation. Conversely, the anterior cingulate thinning was independent of lung function suggesting a direct effect from *DEPTOR* variant expression in view of the known strong *DEPTOR* gene expression in the anterior cingulate cortex.^47^

Our study builds upon our previous work^5^ we used expression quantitative trait loci (eQTL) databases to identify potential genes of interest for IPF. We found the IPF risk signal co-localised with an expression of *DEPTOR* in a very large blood eQTL dataset, a large lung eQTL dataset, and multiple tissues in Genotype-Tissue Expression (GTEx) (including the lung). In addition, it has been also shown that the *RP11-760H22.2*, *TAF2*, and *KB-1471A8.1* co-localised with the IPF risk signal in GTEx (but not the other larger eQTL databases). Although we would never be able to completely rule out the variants acting through another gene, given the variants were located in *DEPTOR*, *DEPTOR* had the strongest evidence for gene expression in relevant tissue and had strong biological supporting evidence,^48^ we felt *DEPTOR* was the most likely gene of interest and was selected as the IPF variant in this locus.

*DEPTOR* in chromosome 8 encodes for the Dishevelled, Egl-10 and Pleckstrin (DEP) domain-containing mechanistic target of rapamycin [mTOR]-interacting protein, which is a key modulator (partial inhibitor) of the mTOR pathways interacting with both of its complexes (mTORC1 and mTORC2),^49^ such that higher levels of *DEPTOR* lead to decreased mTOR activity.^50^ In just over a decade since the discovery of *DEPTOR*, major roles have already been established in cancer, metabolism, and immunity explained by its ubiquitous tissue expression and modulation of fundamental cellular processes. In IPF, the recently identified *DEPTOR* IPF risk allele together with decreased gene expression in lung tissue^5^ highlights a risk mechanism through the induction of profibrogenic phenotypes linked to mTORC1 signalling.^48^ Recent evidence supports that carefully targeted inhibition of the transforming growth factor [TGF]*β*1-mTORC1 axis may hold considerable promise as an anti-fibrotic strategy with the potential to impact multiple IPF pathomechanisms.^51^ Interestingly, *DEPTOR* inhibition was also found in brain tissue in Alzheimer's Disease^52^ and the *DEPTOR*/mTOR ratio is considered to regulate the neuroprotection/neurodegeneration balance in pro-inflammatory states via autophagy regulation. Moreover, *DEPTOR* central nervous system (CNS) expression in the hypothalamus, circumventricular organs, and autonomic nervous regions support increasing evidence for a further critical role in brain-body homeostatic control.^47^

Our analysis provides exciting insights that *DEPTOR* dysregulation through the IPF risk variant is linked with cortical thinning in the anterior cingulate and microstructural changes of major white matter tracts. Moreover, our mediation analysis hints to potential direct *DEPTOR* and indirect lung phenotypic effects using forced vital capacity (FVC), as an established lung function marker in IPF.^53^ The observed IPF risk variant association with distinct cortical thinning in the caudal anterior cingulate did not show FVC mediation and is spatially co-localised with high *DEPTOR* bulk gene expression in the Allen Human Brain Atlas.^46^ This is intriguing, as the anterior cingulate cortex (ACC) is involved in the regulation of emotion processing, and cognitive and emotional impairments are frequent among IPF patients.^13^^,^^21^ Neuroimaging studies have consistently identified structural and functional changes within the anterior cingulate cortex associated with major depression.^54^, ^55^, ^56^, ^57^ This may provide a molecular mechanism to explain the clinical observation of CNS comorbidities in IPF, especially the common anxiety, depression,^23^ and cognitive decline.^21^ It is conceivable that the IPF risk *DEPTOR* variant will also reduce *DEPTOR* expression in areas of high expression, such as the anterior cingulate, resulting in the observed cortical thinning. In turn, the affection of the anterior cingulate may well explain the development of neuropsychiatric comorbidities as evidenced by a large body of neuroimaging studies.^58^

In addition, we propose a second indirect mechanism for the associations found between the chromosome 8 IPF risk variant and more widespread changes in major white matter tracts with potential mediation through FVC. Even if the mediation results were weak, the plausibility of such mechanism cannot be ruled out. Particularly, when considering that the specific characteristics of these imaging associations point to a neuroinflammatory signature that may relate to chronic systemic pro-inflammatory state or tissue hypoxia. High ICVF and reduced diffusivity measures that were correlated with the presence of IPF variant in chromosome 8 correspond to findings in acute neuroinflammation and injuries,^59^ such as traumatic brain injury (TBI), axonal injury, and possible hypoxic swelling. Even if some mechanisms may be shared (e.g., microglial activation), it is unlikely that the white matter microstructure patterns we observe here reflect an acute mechanism of inflammation, but instead, we propose chronic low-level neuroinflammation combined with reduced tissue oxygen levels. Animal models of stress/hypoxia have shown reduced diffusivity in WM (e.g., increased fractional anisotropy and reduced mean and radial diffusivity after 2 weeks of stress,^60^ and reduced diffusivity values in early hypoxia^61^). Notably, reductions of (radial) diffusivity can lead to apparent increases in ICVF, as estimated by the NODDI model.^62^ This model assumes constant diffusivities, so ICVF can be overestimated when diffusivity (assumed constant in the NODDI model) is lower than assumed. The pattern of higher FA/ICVF and lower diffusivity is also consistent with a previous study^32^ after adjusting the risk and reference alleles of the IPF variants (for instance https://open.win.ox.ac.uk/ukbiobank/big40/pheweb33k/variant/8:120934126-A-G). Taken together, hypoxia and chronic neuroinflammation are plausible mechanisms for the observed pattern of subcortical changes in diffusivity.

We detected conspicuously strong effects of IPF genetic risk on brain structure for chromosome 17 that are noteworthy, despite the inconclusive co-localisation analysis. The subcortical endophenotypic pattern of the chromosome 17 IPF risk variant was broadly similar to the microstructural changes seen for the *DEPTOR* risk variant and also overlaps spatially suggesting a similar indirect pathomechanism warranting further mechanistic and mediation studies. Interestingly, the cortical chromosome 17 risk variant signature showed a distinct spatial profile and displayed a dichotomy with cortical thinning but increased cortical surface area. Such a pattern has been reported in a GWAS study, which confirmed the ontogenic dichotomy between thickness and surface area.^63^ The nature of these dissociated cortical changes remains unknown but has been linked to putative neurodevelopmental differences^64^ as cortical thickness and area show very little genetic correlation.^63^ However, there were also differences with the findings in^63^ (e.g., absence of an association between cortical features with the *DEPTOR* risk variant). Direct comparisons between our findings and the ones reported in^63^ are challenging, due to differences in the designs and analyses. For instance, it is unclear how neuroimaging features were de-confounded and harmonised in,^63^ where data were pooled together from 60 different sites with different scanning protocols, while UK Biobank reflects a single pre-harmonised cohort with significant de-confounding, ensuring low-interference from inter-site effects.^65^

Our results showed no association between IPF genetic risk variants and functional imaging features (e.g., functional networks and connectivity). This is not surprising and agrees with previous studies,^32^^,^^66^ where structural and microstructural features have been shown to associate with genetics at more loci than functional features. The exact reasons can be only speculated and can include from higher levels of noise and lower reproducibility in fMRI-extracted features to the fact that more complex models than univariate associations may be needed to capture a less direct relationship between genes and higher-order features that characterise function and cognition. This is further supported by,^66^ which shows that imaging-derived phenotypes related to structure and microstructure (WM tract ICVF, cortical grey-white contrast, WM tract FA, and WM tract diffusivity) are more heritable than ones related to rest/task fMRI. Nevertheless, inclusion of the functional brain endophenotypes in our BWAS analysis was motivated by the known rfMRI-behavioural associations for some of the prominent comorbidities. Hence, with the power of the UK Biobank, it made sense to explore for premanifest endophenotypic gene variant associations. Also, a number of IPF genetic loci that we considered in our analyses were not included in previous studies.^32^^,^^66^ Finally, even excluding the functional features, all observed BWAS associations would have still been valid and in fact more emergent. So, in practice, our reported associations are more on the conservative side.

A limitation of our study is that the potential partial mediations did not survive Bonferroni multiple comparison correction, they were only nominally significant. In the mediation analysis, we used an absolute FVC measure (in litres) and accounted for age, sex, height, and ethnicity (our study cohort is restricted to white European ethnic background). We found strong covariance structure between the 17 IDPs used (i.e., the ones that had significant associations with *DEPTOR* and showed co-localisation with IPF), meaning that the effective degrees of freedom are much lower than 17 (14 of the 17 IDPs show correlations above 0.7 with at least one of the rest—see Supplementary Figure S6), therefore a Bonferroni correction would be too conservative. Nevertheless, we consider these results as preliminary warranting replication. Another limitation is the lack of prior evidence that *DEPTOR* gene expression levels change in the anterior cingulate cortex in association with the IPF risk variant, revealed by the absence of a relevant eQTL signal in the GTEx portal. Whilst this may counter the hypothesis that there is a direct effect of *DEPTOR* on cortical thickness, the GTEx brain cortex analysis may not have sufficient power to identify an effect, as brain cortex samples were amongst the smallest compared with other tissues profiled in GTEx (https://gtexportal.org/home/tissueSummaryPage#donorInfo).

In summary, we exploited imaging and genetic data from more than 32,000 participants available through the UK Biobank population-level resource to explore links between IPF genetic risk and imaging-derived brain endophenotypes. We identified strong associations between cortical thickness (in the anterior cingulate) and white matter microstructure (in major white matter tracts, such as the corpus callosum and corona radiata) with IPF risk loci in chromosome 8 (*DEPTOR* gene) and chromosome 17 (*17q21.31*). Through co-localisation a shared causal gene locus was identified for the *DEPTOR* variant and associated brain signature, and for cingulate cortical thinning no mediation effect from lung function was found. Taken together, these data support the hypothesis that genetic risk profiles may explain some of the observed comorbidity of IPF and brain disorders with further mechanistic studies warranted to characterise additional indirect effects.

## Contributors

AM: data curation, methodology, formal analysis, writing – original draft, RJA: methodology, formal analysis, writing – review & editing, LMK: methodology, writing – review & editing, OCL: resources, writing – review & editing, RGJ: conceptualisation, funding acquisition, writing – review & editing, LVW: conceptualisation, methodology, funding acquisition, writing – review & editing, DPA: supervision, conceptualisation, funding acquisition, writing – original draft, SNS: methodology, resources, supervision, funding acquisition, conceptualisation, writing – original draft. AM, RJA, LMK, and SNS have accessed and verified all the underlying data. AM, RGJ, DPA, and SNS were responsible for the decision to submit the manuscript. All authors read and approved the final version of the manuscript.

## Data sharing statement

All data used in this study are publicly available from the UK Biobank (www.ukbiobank.ac.uk). Our study was performed under UK Biobank Project 43822.

Analysis code will be available on GitHub (github.com/Alirezamnk/BWAS_IPF) at the time of publication.

## Declaration of interests

RGJ reports research funding from AstraZeneca, Biogen, Galecto, GlaxoSmithKline, RedX, Pliant, and Genetech; personal fees from Bristol Myers Squibb, Daewoong, Veracyte, Resolution Therapeutics, RedX, Pliant, Chiesi, Roche, PatientMPower, AstraZeneca, GSK, Boehringer Ingelheim, Galapagos, and Vicore; non-financial support from NuMedii and Action for Pulmonary Fibrosis, outside the submitted work. LVW reports research funding from GSK and Orion Pharma and have conducted consultancy for Galapagos. DPA reports research funding from Biogen. The other authors have no conflict of interest to disclose.

## Appendix A Supplementary data

Supplementary Material

DEMISTIFI Consortium docx

## References

[1] Strongman H., Kausar I., Maher T.M. (2018). Incidence, prevalence, and survival of patients with idiopathic pulmonary fibrosis in the UK. Adv Ther.

[2] Richeldi L., Collard H.R., Jones M.G. (2017). Idiopathic pulmonary fibrosis. Lancet.

[3] Kim H.J., Perlman D., Tomic R. (2015). Natural history of idiopathic pulmonary fibrosis. Respir Med.

[4] Raghu G., Collard H.R., Egan J.J. (2011). An official ATS/ERS/JRS/ALAT statement: idiopathic pulmonary fibrosis: evidence-based guidelines for diagnosis and management. Am J Respir Crit Care Med.

[5] Allen R.J., Guillen-Guio B., Oldham J.M. (2020). Genome-wide association study of susceptibility to idiopathic pulmonary fibrosis. Am J Respir Crit Care Med.

[6] Noth I., Zhang Y., Ma S.-F. (2013). Genetic variants associated with idiopathic pulmonary fibrosis susceptibility and mortality: a genome-wide association study. Lancet Respir Med.

[7] Allen R.J., Porte J., Braybrooke R. (2017). Genetic variants associated with susceptibility to idiopathic pulmonary fibrosis in people of European ancestry: a genome-wide association study. Lancet Respir Med.

[8] Fingerlin T.E., Murphy E., Zhang W. (2013). Genome-wide association study identifies multiple susceptibility loci for pulmonary fibrosis. Nat Genet.

[9] Luppi F., Kalluri M., Faverio P., Kreuter M., Ferrara G. (2021). Idiopathic pulmonary fibrosis beyond the lung: understanding disease mechanisms to improve diagnosis and management. Respir Res.

[10] Mathai S.K., Newton C.A., Schwartz D.A., Garcia C.K. (2016). Pulmonary fibrosis in the era of stratified medicine. Thorax.

[11] Ley B., Collard H. (2013). Epidemiology of idiopathic pulmonary fibrosis. Clin Epidemiol.

[12] Makarev E., Izumchenko E., Aihara F. (2016). Common pathway signature in lung and liver fibrosis. Cell Cycle.

[13] Bors M., Tomic R., Perlman D.M., Kim H.J., Whelan T.P. (2015). Cognitive function in idiopathic pulmonary fibrosis. Chron Respir Dis.

[14] Tudorache V., Traila D., Marc M. (2019). Impact of moderate to severe obstructive sleep apnea on the cognition in idiopathic pulmonary fibrosis. PLoS One.

[15] Mahammedi A., Ramos A., Bargalló N. (2021). Brain and lung imaging correlation in patients with COVID-19: could the severity of lung disease reflect the prevalence of acute abnormalities on neuroimaging? A global multicenter observational study. Am J Neuroradiol.

[16] Chacón-Aponte A.A., Durán-Vargas É.A., Arévalo-Carrillo J.A. (2022). Brain-lung interaction: a vicious cycle in traumatic brain injury. Acute Crit Care.

[17] Tudorache E., Marc M., Traila D., Manolescu D. (2020). Cognitive impairment in chronic lung diseases. An overview and management of multiple chronic conditions. IntechOpen.

[18] Roy B., Woo M.S., Vacas S., Eshaghian P., Rao A.P., Kumar R. (2021). Regional brain tissue changes in patients with cystic fibrosis. J Transl Med.

[19] Dodd J.W. (2015). Lung disease as a determinant of cognitive decline and dementia. Alzheimer's Res Ther.

[20] Li C., Chen W., Lin F. (2022). Functional two-way crosstalk between brain and lung: the brain–lung axis. Cell Mol Neurobiol.

[21] Lutsey P.L., Chen N., Mirabelli M.C. (2019). Impaired lung function, lung disease, and risk of incident dementia. Am J Respir Crit Care Med.

[22] Hubbard R.B., Smith C., Le Jeune I., Gribbin J., Fogarty A.W. (2008). The association between idiopathic pulmonary fibrosis and vascular disease: a population-based study. Am J Respir Crit Care Med.

[23] Holland A.E., Fiore J.F., Bell E.C. (2014). Dyspnoea and comorbidity contribute to anxiety and depression in interstitial lung disease. Respirology.

[24] Rose E.J., Donohoe G. (2013). Brain vs behavior: an effect size comparison of neuroimaging and cognitive studies of genetic risk for schizophrenia. Schizophr Bull.

[25] Meyer-Lindenberg A., Weinberger D.R. (2006). Intermediate phenotypes and genetic mechanisms of psychiatric disorders. Nat Rev Neurosci.

[26] Miller K.L., Alfaro-Almagro F., Bangerter N.K. (2016). Multimodal population brain imaging in the UK Biobank prospective epidemiological study. Nat Neurosci.

[27] Bookheimer S.Y., Strojwas M.H., Cohen M.S. (2000). Patterns of brain activation in people at risk for Alzheimer's disease. N Engl J Med.

[28] Bycroft C., Freeman C., Petkova D. (2018). The UK Biobank resource with deep phenotyping and genomic data. Nature.

[29] Alfaro-Almagro F., Jenkinson M., Bangerter N.K. (2018). Image processing and quality control for the first 10,000 brain imaging datasets from UK Biobank. Neuroimage.

[30] Manichaikul A., Mychaleckyj J.C., Rich S.S., Daly K., Sale M., Chen W.-M. (2010). Robust relationship inference in genome-wide association studies. Bioinformatics.

[31] Alfaro-Almagro F., McCarthy P., Afyouni S. (2021). Confound modelling in UK Biobank brain imaging. Neuroimage.

[32] Elliott L.T., Sharp K., Alfaro-Almagro F. (2018). Genome-wide association studies of brain imaging phenotypes in UK Biobank. Nature.

[33] Cai J.-F., Candès E.J., Shen Z. (2010). A Singular value thresholding algorithm for matrix completion. SIAM J Optim.

[34] Smith S.M., Nichols T.E. (2018). Statistical challenges in “big data” human neuroimaging. Neuron.

[35] Dhindsa R.S., Mattsson J., Nag A. (2021). Identification of a missense variant in SPDL1 associated with idiopathic pulmonary fibrosis. Commun Biol.

[36] Dressen A., Abbas A.R., Cabanski C. (2018). Analysis of protein-altering variants in telomerase genes and their association with MUC5B common variant status in patients with idiopathic pulmonary fibrosis: a candidate gene sequencing study. Lancet Respir Med.

[37] Chang C.C., Chow C.C., Tellier L.C., Vattikuti S., Purcell S.M., Lee J.J. (2015). Second-generation PLINK: rising to the challenge of larger and richer datasets. GigaScience.

[38] Buuren S van, Groothuis-Oudshoorn K. (2011). Mice: multivariate imputation by chained equations in R. J Stat Softw.

[39] Thompson A., Pirmohamed M. (2021). Associations between occupation and heavy alcohol consumption in UK adults aged 40–69 years: a cross-sectional study using the UK Biobank. BMC Public Health.

[40] Giambartolomei C., Vukcevic D., Schadt E.E. (2014). Bayesian test for colocalisation between pairs of genetic association studies using summary statistics. PLoS Genet.

[41] Gold W.M., Koth L.L. (2016).

[42] Sobel M.E. (1982). Asymptotic confidence intervals for indirect effects in structural equation models. Sociol Methodol.

[43] Giambartolomei C., Zhenli Liu J., Zhang W. (2018). A Bayesian framework for multiple trait colocalization from summary association statistics. Bioinformatics.

[44] Boettger L.M., Handsaker R.E., Zody M.C., McCarroll S.A. (2012). Structural haplotypes and recent evolution of the human 17q21.31 region. Nat Genet.

[45] Wells A.U. (2013). Forced vital capacity as a primary end point in idiopathic pulmonary fibrosis treatment trials: making a silk purse from a sow's ear. Thorax.

[46] Hawrylycz M.J., Lein E.S., Guillozet-Bongaarts A.L. (2012). An anatomically comprehensive atlas of the adult human brain transcriptome. Nature.

[47] Caron A., Briscoe D.M., Richard D., Laplante M. (2018). DEPTOR at the nexus of cancer, metabolism, and immunity. Physiol Rev.

[48] Woodcock H.v., Eley J.D., Guillotin D. (2019). The mTORC1/4E-BP1 axis represents a critical signaling node during fibrogenesis. Nat Commun.

[49] Catena V., Fanciulli M. (2017). Deptor: not only a mTOR inhibitor. J Exp Clin Cancer Res.

[50] Peterson T.R., Laplante M., Thoreen C.C. (2009). DEPTOR is an mTOR inhibitor frequently overexpressed in multiple myeloma cells and required for their survival. Cell.

[51] Platé M., Guillotin D., Chambers R.C. (2020). The promise of mTOR as a therapeutic target pathway in idiopathic pulmonary fibrosis. Eur Respir Rev.

[52] Davies J., Zachariades E., Rogers-Broadway K.-R., Karteris E. (2014). Elucidating the role of DEPTOR in Alzheimer's disease. Int J Mol Med.

[53] Young R.P., Hopkins R., Eaton T.E. (2007). Forced expiratory volume in one second: not just a lung function test but a marker of premature death from all causes. Eur Respir J.

[54] Greicius M.D., Flores B.H., Menon V. (2007). Resting-state functional connectivity in major depression: abnormally increased contributions from subgenual cingulate cortex and thalamus. Biol Psychiatry.

[55] Mayberg H.S., Lozano A.M., Voon V. (2005). Deep brain stimulation for treatment-resistant depression. Neuron.

[56] Caetano S.C., Kaur S., Brambilla P. (2006). Smaller cingulate volumes in unipolar depressed patients. Biol Psychiatry.

[57] Wagner G., Schultz C.C., Koch K., Schachtzabel C., Sauer H., Schlösser R.G. (2012). Prefrontal cortical thickness in depressed patients with high-risk for suicidal behavior. J Psychiatr Res.

[58] Chen Y., Dang M., Zhang Z. (2021). Brain mechanisms underlying neuropsychiatric symptoms in Alzheimer's disease: a systematic review of symptom-general and –specific lesion patterns. Mol Neurodegener.

[59] Kamiya K., Hori M., Aoki S. (2020). NODDI in clinical research. J Neurosci Methods.

[60] Magalhães R., Bourgin J., Boumezbeur F. (2017). White matter changes in microstructure associated with a maladaptive response to stress in rats. Transl Psychiatry.

[61] Tao J.D., Barnette A.R., Griffith J.L., Neil J.J., Inder T.E. (2012). Histopathologic correlation with diffusion tensor imaging after chronic hypoxia in the immature ferret. Pediatr Res.

[62] Lampinen B., Szczepankiewicz F., Mårtensson J., van Westen D., Sundgren P.C., Nilsson M. (2017). Neurite density imaging versus imaging of microscopic anisotropy in diffusion MRI: a model comparison using spherical tensor encoding. Neuroimage.

[63] Grasby K.L., Jahanshad N., Painter J.N. (2020). The genetic architecture of the human cerebral cortex. Science (1979).

[64] Winkler A.M., Kochunov P., Blangero J. (2010). Cortical thickness or grey matter volume? The importance of selecting the phenotype for imaging genetics studies. Neuroimage.

[65] Perkonigg M., Hofmanninger J., Herold C.J. (2021). Dynamic memory to alleviate catastrophic forgetting in continual learning with medical imaging. Nat Commun.

[66] Smith S.M., Douaud G., Chen W. (2021). An expanded set of genome-wide association studies of brain imaging phenotypes in UK Biobank. Nat Neurosci.

