# Supplementary material for: Mapping brain endophenotypes associated with idiopathic pulmonary fibrosis genetic risk
Source: eBioMedicine. 2022 Nov 19;86:104356. doi: 10.1016/j.ebiom.2022.104356 (PMC9677133; doi:10.1016/j.ebiom.2022.104356)
Supplement: DEMISTIFI Consortium docx [file mmc2.docx]

**DEMISTIFI Consortium Membership**

| **No.** | **First Name** | **Surnames** | **affiliations** |
| --- | --- | --- | --- |
| 1 | Ali-Reza | Mohammadi-Nejad | Sir Peter Mansfield Imaging Centre & Mental Health and Clinical Neurosciences, School of Medicine, University of Nottingham, Nottingham, UK |
| 2 | Richard J. | Allen | Department of Health Sciences, University of Leicester, Leicester, UK |
| 3 | Luke M. | Kraven | Department of Health Sciences, University of Leicester, Leicester, UK |
| 4 | Olivia C. | Leavy | Department of Health Sciences, University of Leicester, Leicester, UK |
| 5 | R. Gisli | Jenkins | National Heart and Lung Institute, Imperial College London, London, UK |
| 6 | Louise V. | Wain | Department of Health Sciences, University of Leicester, Leicester, UK |
| 7 | Dorothee P. | Auer | Sir Peter Mansfield Imaging Centre & Mental Health and Clinical Neurosciences, School of Medicine, University of Nottingham, Nottingham, UK |
| 8 | Stamatios N. | Sotiropoulos | Sir Peter Mansfield Imaging Centre & Mental Health and Clinical Neurosciences, School of Medicine, University of Nottingham, Nottingham, UK |
| 9 | Jennifer K. | Quint | National Heart and Lung Institute, Imperial College London, London, UK |
| 10 | Iain D. | Stewart | National Heart and Lung Institute, Imperial College London, London, UK |
| 11 | Rachel | Chambers | Centre for Inflammation and Tissue Repair, University College London, London, UK |
| 12 | Maria | Kaisar | Nuffield Department of Surgical Sciences, University of Oxford, Oxford, UK |
| 13 | Rutger J. | Ploeg | Nuffield Department of Surgical Sciences, University of Oxford, Oxford, UK  Biomedical Research Centre Oxford, Oxford, UK |
| 14 | Guruprasad P. | Aithal | NIHR Nottingham BRC, Nottingham University Hospitals NHS Trust and the University of Nottingham, Nottingham, UK |
| 15 | Nicholas | Selby | Centre for Kidney Research and Innovation, School of Medicine, University of Nottingham, Nottingham, UK |
| 16 | Gordon W. | Moran | NIHR Nottingham BRC, Nottingham University Hospitals NHS Trust and the University of Nottingham, Nottingham, UK |
| 17 | Neil | Guha | NIHR Nottingham BRC, Nottingham University Hospitals NHS Trust and the University of Nottingham, Nottingham, UK |
| 18 | Simon | Johnson | NIHR Nottingham BRC, Nottingham University Hospitals NHS Trust and the University of Nottingham, Nottingham, UK |
| 19 | Tom | Giles | The Digital Research Service & The Advanced Data Analysis Centre, University of Nottingham, Nottingham, UK |
| 20 | Philip | Quinlan | The Digital Research Service, University of Nottingham, Nottingham, UK |
| 21 | Lisa | Chakrabarti | School of Veterinary Medicine and Science, University of Nottingham |
| 22 | Xin | Chen | School of Computer Science, University of Nottingham, UK |
| 23 | Susan | Francis | Sir Peter Mansfield Imaging Centre, School of Physics, University of Nottingham, Nottingham, UK |
| 24 | Fasihul | Khan | Respiratory Medicine, School of Medicine, University of Nottingham, Nottingham, UK |
| 25 | Karen | Piper Hanley | Division of Gastroenterology and Hepatology, Manchester University NHS Foundation Trust, Manchester, UK |
| 26 | Chris | Scotton | Medical School, University of Exeter, Exeter, UK |
| 27 | Hilary | Longhurst | Dyskeratosis Congenita (DC) Action, UK |
| 28 | Jane | Paxton | Dyskeratosis Congenita (DC) Action, UK |
| 29 | Rob | Slack | Galecto, Stevenage, Hertfordshire, UK |
| 30 | Wendy | Adams | Action for Pulmonary Fibrosis, Peterborough, UK |
| 31 | Anna | Duckworth | EPIC, Exeter Patients in Collaboration for pulmonary Fibrosis, Exeter, UK |
| 32 | Scott | Turner | Pliant Therapeutics, San Francisco, CA, USA |
